# Walking on Virtual Surface Patterns Leads to Changed Control Strategies

**DOI:** 10.3390/s24165242

**Published:** 2024-08-13

**Authors:** Maximilian Stasica, Celine Honekamp, Kai Streiling, Olivier Penacchio, Loes van Dam, André Seyfarth

**Affiliations:** 1 Lauflabor Locomotion Laboratory, Institute of Sports Science and Centre for Cognitive Science, Technical University of Darmstadt, 64289 Darmstadt, Germany; 2 Sensorimotor Control and Learning Lab, Centre for Cognitive Science, Technical University of Darmstadt, 64289 Darmstadt, Germany; 3 Computer Science Department, Universitat Autònoma de Barcelona, 08193 Barcelona, Spain

**Keywords:** human gait, biomechanics, architecture, perception, pattern, illusion, virtual reality (VR), inclusive design, structural design, visual perturbations

## Abstract

Inclusive design does not stop at removing physical obstacles such as staircases. It also involves identifying architectural features that impose sensory burdens, such as repetitive visual patterns that are known to potentially cause dizziness or visual discomfort. In order to assess their influence on human gait and its stability, three repetitive patterns—random dots, repetitive stripes, and repetitive waves (Lisbon pattern)—were displayed in a coloured and greyscale variant in a virtual reality (VR) environment. The movements of eight participants were recorded using a motion capture system and electromyography (EMG). During all test conditions, a significant increase in the muscular activity of leg flexor muscles was identified just before touchdown. Further, an increase in the activity of laterally stabilising muscles during the swing phase was observed for all of the test conditions. The lateral and vertical centre of mass (CoM) deviation was statistically evaluated using a linear mixed model (LMM). The patterns did cause a significant increase in the CoM excursion in the vertical direction but not in the lateral direction. These findings are indicative of an inhibited and more cautious gait style and a change in control strategy. Furthermore, we quantified the induced discomfort by using both algorithmic estimates and self-reports. The Fourier-based methods favoured the greyscaled random dots over repetitive stripes. The colour metric favoured the striped pattern over the random dots. The participants reported that the wavey Lisbon pattern was the most disruptive. For architectural and structural design, this study indicates (1) that highly repetitive patterns should be used with care in consideration of their impact on the human visuomotor system and its behavioural effects and (2) that coloured patterns should be used with greater caution than greyscale patterns.

## 1. Introduction

This paper is an extension of the conference paper [1] that is also included in [2]. It adds kinematic data, a more in-depth analysis of muscular activity, establishes a connection with existing biomechanical theories, and compares the results to algorithmic estimates of visual discomfort.

Quality of life is heavily influenced by mobility and health—especially in the elderly. As populations age [3,4], the number of people with limitations and disabilities continues to increase. According to a survey from the German Institute for Applied Social Sciences, the proportion of people with disability status has risen by 9% within the last 8 years [5]. Currently, 9.4% of the German population has the status of being “severely disabled” [6], which corresponds to 7.8 million people in Germany alone. The World Health Organisation (WHO) estimates that 1.3 billion people worldwide, or 16% of the global population, are affected by disabilities [7].

The United States Centers for Disease Control and Prevention defines disabilities with a specific focus on activity limitation and participation restriction [8], highlighting the importance of designing environments that allow for the participation and activity of all members of society. However, in urban environments, we are often confronted with architectural design properties that focus on aesthetics rather than function or inclusive design. These design features often contain repetitive patterns (e.g., through either colouring or lighting and shadows), which are known to possibly induce perceptual perturbations, such as optical illusions or discomfort, especially in people with physiological and psychological disorders [9,10], which can hinder their participation and activity.

Such perceptual perturbations come in a variety of forms and can be caused by different environmental aspects. For example, flickering light, e.g., when walking through light and shadow patterns, has been shown to trigger a variety of symptoms in people who experience migraines, epilepsy, or strong photosensitivity [9]. Photophobia or increased photosensitivity causes strong negative responses to bright stimuli, intermittent light sources, complex brightness stimuli, and particularly flickering lights or high-contrast patterns [11]. For some people, visual patterns may also cause mismatches between visual and vestibular information, which, in turn, can affect their balance and gait [12] or lead to motion or cyber sickness [13,14,15,16]. Moreover, misinterpretations of surroundings, such as wrongly perceived barriers, obstructions, curbs, or the edges of steps, can lead to imbalance, even in participants without any particular disorder [9]. Discomfort glare can also cause visual fatigue [12]. Glare is a visual difficulty experienced in the presence of light that is significantly brighter than what the eyes have adjusted to [12]. Such effects can arise both due to artificial light and daylight and have to be avoided during the design of architectural structures [12]. As proposed in [17], psychological stress, which can be induced by visual discomfort, can even lead to the evaluation of alternative routes to avoid the structure causing discomfort. It is, therefore, in the best interest of structural designers and architects to evaluate design proposals based on both objective and subjective measures for inclusive design. Biomechanical parameters might possibly be such an objective measure. While the connection between emotional states, postural control, and gait style has been thoroughly explored for acrophobia (e.g., [18,19,20,21]), it has only been sparsely covered with regard to visual patterns. A notable example can be found in [22], where it was concluded that patterns on the floor could lead to a deviation from the desired movement direction, and the work in [23] first described a moving room illusion, in which externally generated optical flow causes balance problems in all age groups.

**Figure 1:**
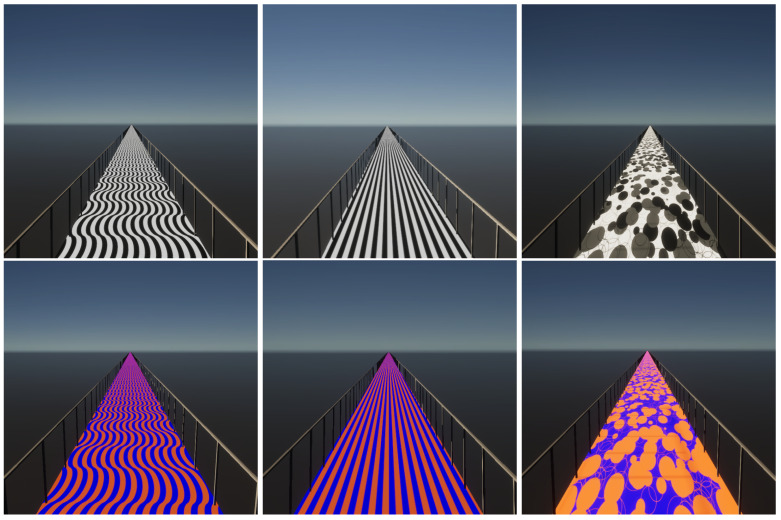
Overview of the patterns used in this experiment. The Lisbon pattern is shown in the left column, the striped pattern is in the middle column, and the random pattern is in the right column. Each of these patterns was displayed either in greyscale or colour (blue Hex #0000ff and orange Hex #ff6600). Participants uniformly reported the Lisbon pattern to be the most uncomfortable to walk on. For simplification, the bridges are presented with a neutral background.

Optical flow refers to the change in a visual image that falls on the retina [24], which is either externally generated by the motion of objects around the observer, e.g., cars, trains, advertisements, or people, or by the motion of the observer through the environment. Humans rely on optical flow to perceive motion visually. Patterns due to colours or lighting can influence this perceived motion and cause difficulties in terms of distinguishing between externally caused optical flow and optical flow due to self-motion [24]. As demonstrated by the moving room illusion, optical flow is important to human balancing [23] and directly influences body sway [25]. It is especially important for the elderly or patients with balancing problems such as those caused by Parkinson’s disease [26]. Further examples of adverse visual stimuli, often found in man-made environments, include the Lisbon (see Figure 1) and café-wall patterns [10] or the aperture illusion [22,27] due to partial exclusion. The authors of [28] discuss additional visual stimuli in built environments, which can cause balance issues or affect well-being, especially for neurodivergent people. In short, a variety of evidence exists that visual patterns in the environment can affect human interaction with the environment, both in groups with particular disorders and the general population. However, the manifestation of visual discomfort in human gait characteristics remains to be quantified.

In order to address this gap, we investigate the influence of repetitive floor surface patterns on human gait using virtual reality (VR).

In recent years, many researchers have investigated the effect of VR on human gait. Their key findings are a significant decrease in stride length for free walking [29] and treadmill walking [30,31], an increase in step width [30] and rates of weight acceptance force, as well as push-off force [32], and an increase in cadence [31]. Ref. [30] also found an increase in the variability of stride velocity and step width. Conflicting evidence was provided in [33], in which the said increase in variability was not identified. Since the methods and procedures of [30,33] differed, no clear conclusion can be drawn. Further, an increase in the centre of pressure of an ellipse area was observed, which is indicative of the stability problems of the participants [31]. However, VR can have manifold effects on human gait even without any manipulations, and the ability to control the visual environment makes it a worthwhile option for gait experiments. If optical flows are induced through VR, this can lead to a further reduced step length, increased step width, and higher stride variability [34]. Again, ref. [33] came to the conclusion that the amount and structure of the variability are not significantly affected.

By taking the findings from previous studies [1,2,29] into account, we, therefore, hypothesise that repetitive patterns inducing strong optical flow promote a more cautious and inhibited gait style, as reflected in an increased lateral and vertical excursion of the body’s centre of mass (CoM) and changes in muscular activity.

## 2. Materials and Methods

### 2.1. Participants

A total of 12 participants (five female and seven male) with either normal or corrected-to-normal vision took part in the experiment. They were between 19 and 33 years of age (μ=23.42; median =22; σ = 4.25) and ranged from 156 cm to 190 cm in body height (μ=174.0; median =174.5; σ = 9.43). Their body weights were between 50 kg and 89 kg (μ=67.83; median =67.0; σ = 12.78). None of the participants had any physical disabilities, and two reported a fear of heights. Six participants reported having no previous experience with VR.

In total, four participants were excluded due to technical difficulties (excessive displaying of grey screens due to the misalignment of the WiFi link box to the wireless VR set) during the experiment, leaving a dataset of eight participants. In order to test the effects of virtual repetitive surface patterns on human gait, we used an improved version of the VR setup, as presented in [35]. This setup mainly consists of a 5 m long walking track, where participants can walk freely without a treadmill. This is coupled with a motion capture system and an electromyography (EMG) system. The improvements involved more processing power and a wireless head-mounted display (HMD), which increases the immersion while also reducing the risk of stumbling over the HMD cable. We further changed the virtual environment to an urban setting to increase immersion and depth perception. The experiment was conducted in accordance with the Declaration of Helsinki and was approved by the Ethics Board of the Technical University of Darmstadt (EK83/2022).

The participants were placed in an immersive virtual environment, which was built using the Unity development platform [36] and displayed using a VR headset (HTC Vive Pro Eye, Valve, Bellevue, US & HTC, Taoyuan, Taiwan). The VR contained a simple, prebuilt model of a major city with high buildings, roads, and trees and a custom-made, parameterised footbridge, over which the participants had to walk.

The pattern and colour of the 2.5 m wide bridge were varied between trials. All pattern and colour combinations are depicted in Figure 1. First, since it is a simple repetitive pattern, a surface consisting of stripes, which were parallel to each other, was included as an experimental condition, called the “striped pattern” (Figure 1). Further, a surface of waved stripes was included, called the “Lisbon pattern”, since it resembles the calçada portuguesa [37] in Lisbon (Figure 1). For both of these patterns, the spatial frequency of the stripes was set to 5 cycles per meter. Since these patterns are both made of repetitive lines, the third surface, which consisted of random dots called the “random pattern” (Figure 1), was chosen. Both greyscale and coloured versions of each pattern were included. For the coloured version, blue (Hex #0000ff) and orange (Hex #ff6600) were chosen for maximum contrast. For the control condition, the bridge had a uniform grey floor in order to serve as a baseline condition for normal walking, to which the experimental conditions were compared.

The materials applied in VR were specifically nonreflective to avoid lighting artefacts; colour intensity in the virtual environment and on the HMDs was not measured. The bridge was placed in an urban setting to enhance the realism of the scene. Together, this resulted in six experimental conditions and one control condition.

### 2.2. Measurement Equipment

**Figure 2:**
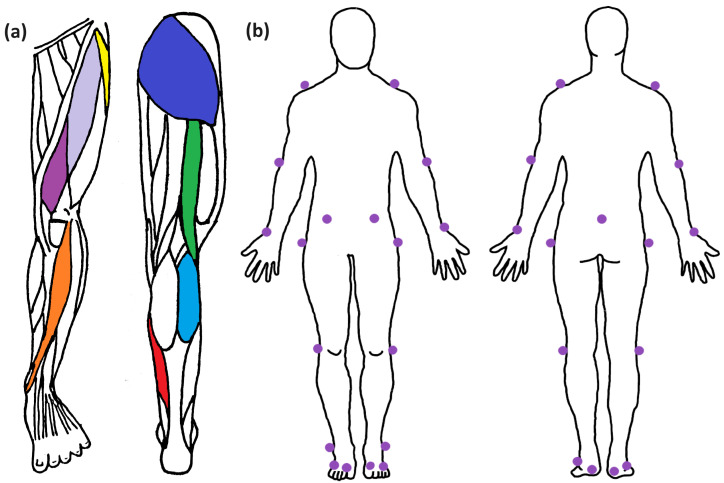
Overview of the sensor and marker placements. (**a**) Shows the leg muscles considered in this experiment. Sensors were placed on the tensor fasciae latae (yellow), rectus femoris (light purple), vastus medialis (dark purple), tibialis anterior (orange), gluteus maximus (dark blue), biceps femoris (green), gastrocnemius lateralis (light blue), and soleus (red). (**b**) Denotes the placements of the passive markers for the motion capture system. A custom-made reduced markerset was used to extract the necessary kinematic data while keeping an efficient experimental protocol. No marker was placed on the C7 chord since its visibility would be mostly blocked by the VR set. Markers are represented by purple dots.

Biomechanical measurement devices were utilised to record the relevant gait parameters. The participants were equipped with 16 wireless surface electromyography (EMG) sensors (Trigno Avanti, Delsys, Natick, MA, USA), which were placed parallel to the muscle fibres on the muscle belly of eight gait-relevant muscles per limb, including the musculus Tibialis Anterior (TIB), m. Soleus (SOL), m. Gastrocnemius Lateralis (GAS), m. Vastus Medialis (VAS), m. Rectus Femoris (RCF), m. Biceps Femoris (BCF), m. Tensor Fasciae Latae (TFL), and m. Gluteus Maximus (GLM) (see Figure 2). Skin preparation included the removal of body hair and cleaning the skin with alcohol. The rectangular electrodes (27 × 37 × 13 mm, mass 14 g) were attached with an adhesive. The sensors feature four silver bar contacts with an intra-electrode distance of 10 mm and collected data with a sampling rate of 1,259 Hz. Each sensor also contained a hardware-integrated inertial measurement unit (IMU) with a 3D gyroscope (148 Hz).

Additionally, movement data were collected using a nine-infrared-camera-based motion capture system (Qualisys Oqus). For this, we used a reduced full body marker set consisting of 21 passive reflective markers. Additionally, the head movement was tracked using the position and rotation of the head-mounted display (HMD) and was logged by the VR system.

### 2.3. Sensor and Marker Placement

In total, 21 passive markers for the motion capture system and 16 EMG electrodes were fitted to each participant.

The EMG equipment was placed on eight muscles on each leg (tensor fasciae latae, rectus femoris, vastus medialis, tibialis anterior, gluteus maximus, biceps femoris, gastrocnemius lateralis, and soleus), as these were the most relevant extensors and flexors for the hip, knee, and ankle joints during gait analysis. Since all of these sensors contain an IMU, further details about the orientation and movement of the leg segments were measured independently of muscle activation.

In order to capture the movements of the participants in more detail, 21 reflective markers were placed at easily identifiable landmarks. The selection of these spots followed a custom-made reduced markerset. From bottom to top, these markers were placed on the proximal joint of the first and fifth toe, the heel, the lateral ankle joint, the lateral knee joint, and the upper end of the femur bone on both legs. The markers were placed on both the anterior superior illiac and the sacrum on the torso. The upper limbs were covered by the shoulder, anterior elbow, and anterior wrist markers. The additional marker that is usually placed at the C7 chord located in the neck region was omitted to prevent entanglement with the VR headset. Since the head position was not the focus of this experiment and could theoretically be measured using the integrated position tracking of the HMD, no head markers were placed.

### 2.4. Measurement Protocol

After preparation, the headset was adjusted for the participant, who was then provided time to adapt to the VR setup. During this period, the participants were allowed to freely explore the SteamVR home and the boundaries of the virtual world with respect to the real room. This longer adaptation time was included to avoid the effect of reduced stride length due to unfamiliarity and insecurity about the surroundings [29]. After the adaptation phase, each participant went through all conditions in a randomised order. They were allowed to freely view their surroundings while walking to ensure a behaviour that was as ecologically valid as possible. Within each condition, the participants performed five trials. Typically, each trial contained three full-gait cycles per leg and an initiation cycle. Since this study focuses on a stable gait, the initiation cycle was neglected in the analysis of the EMG data. Upon nearing the end of the measurement path, the participants were shown a stop sign and a horizontal barrier in VR and were asked to stop in front of it. Every condition featured a virtual bridge and one of the surface patterns depicted in Figure 1, while the baseline condition took place on a neutral grey surface. After the VR session, each participant was asked to report which pattern they felt to be the most uncomfortable to them.

### 2.5. Data Processing

All the data were processed and analysed using Matlab 2023a. The EMG data were processed using the method outlined in [38]. The raw signal was demeaned to eliminate potential offsets, bandpass-filtered (10–450 Hz; fourth-order Butterworth), and time-normalised over one gait cycle. The time-normalised signal was then highpass-filtered (6 Hz; second-order Butterworth) and aggregated over each condition.

The individual strides were identified based on gyroscope-stride identification [38]. In order to do this, gyroscopic data in the sagittal plane from three of the sensors (TIB, SOL, and GAS) integrated into the EMG system were combined to obtain a reliable measure for the orientation of the shank. Following [38], a heel strike was defined using the first zero-passing of the combined angular velocity after a highly negative combined signal. Further, the first stride of each leg was neglected to avoid gait initiation effects. Lastly, the grand mean [38] of each condition was calculated over all participants and used for detailed analysis. In order to check whether any possible changes arising from the pattern or the colour used, 95% confidence bands for continuous data were calculated using the method outlined in [39]. The test conditions significantly differed from the control if they crossed the confidence bands. This representation therefore allows for an easy visual identification of the significant differences between the test conditions and the control.

The motion capture data were postprocessed in Qualisys Track Manager (QTM) (Qualisys, Gothenburg, Sweden) and analysed using Matlab 2023a, The MathWorks Inc., Natick, MA, USA.

In order to eliminate any possible misalignments in the virtual environment, all reported CoM values were adjusted to the direction of walking during each trial. This serves as a fail-safe to ensure the quality of the data.

A linear mixed model (LMM) was calculated to statistically test the impact of the patterns and colouring on lateral and vertical CoM deviation.

### 2.6. Algorithmic Estimation of Visual Disturbance

**Figure 3:**
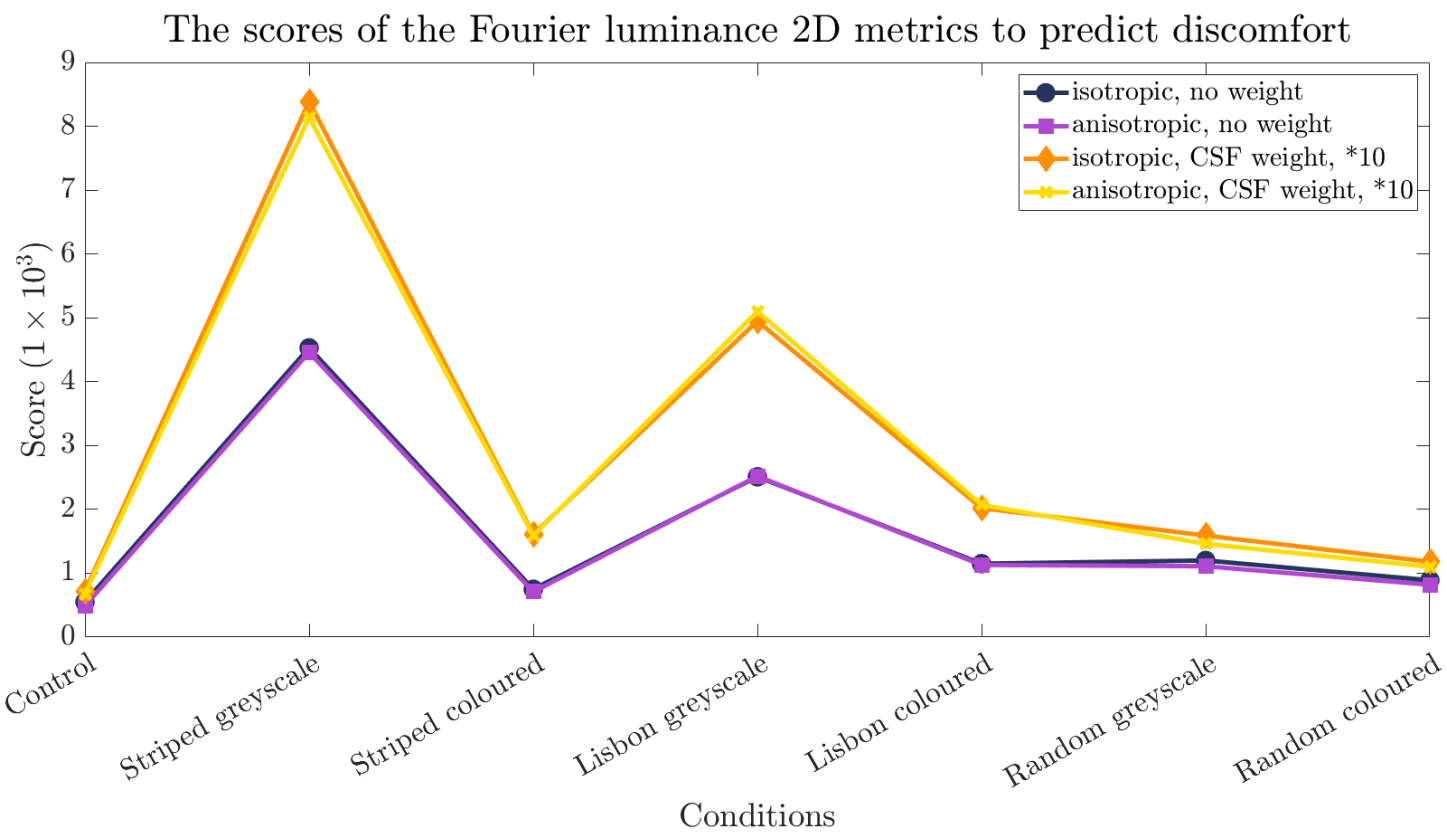
Overview of the scores of all the conditions, assigned by four different Fourier luminance 2D metrics [40]. Note that each metric assigns the highest values to the greyscaled striped and Lisbon patterns. Moreover, note that the metrics featuring the CSF weights are multiplied by 10 for visualisation purposes.

**Figure 4:**
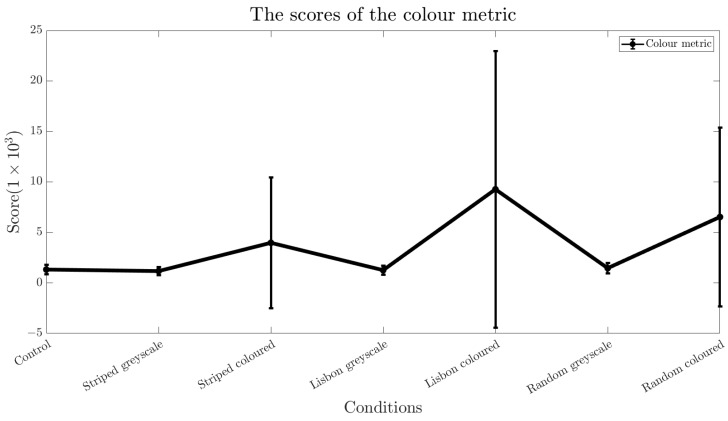
Overview of the scores of the colour metric. As expected, the coloured versions of the patterns scored higher than the greyscaled versions. Further, the highest values are assigned to the coloured Lisbon pattern. Note the large error bars for all the coloured versions.

Additionally, we used algorithmic estimates of visual discomfort [40,41]. The first algorithms are based on the colour content of the images. It either considers an isotropic or anisotropic 1/f cone as the reference for natural images and either weighs the residuals using a contrast sensitivity function (CSF) or applies no weights [40]. The results of the Fourier-based metrics are depicted in Figure 3, with lower scores indicating a lower visual disturbance. The second metric predicts discomfort from the chromatic content of the images, again with lower scores for lower visual discomfort. The results and the respective standard deviations are noted in Figure 4.

## 3. Results

### 3.1. Muscular Activity

**Figure 5:**
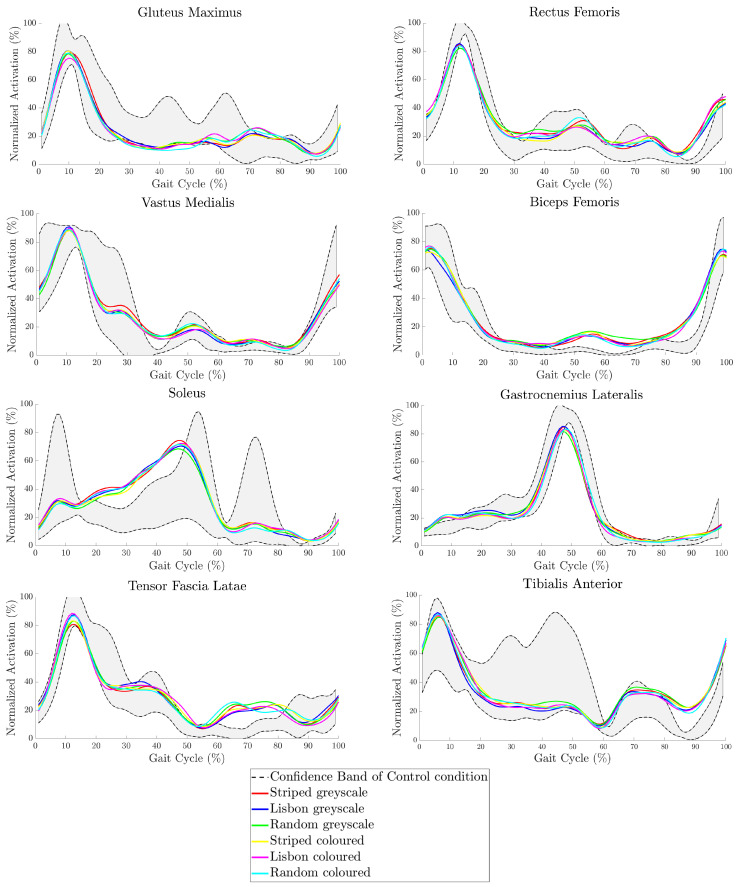
Overview of the grand mean of muscular activation for all participants per condition and muscle. Muscular activation was normalised to the maximum activation during each trial and time-normalised to the gait cycle. The dotted line indicates the 95% confidence interval of the control condition. This implies that each time a line crosses the dotted line, it is significantly different from the control. For the extensor muscles gluteus maximus, vastus medialis, and soleus, this happens around the second half of the stance phase (30–60% of the gait cycle), while the activity of the flexor muscles rectus femoris, biceps femoris, and gastrocnemius lateralis increases significantly during the end of the swing phase (80–90% of the gait cycle). The stabilising tensor fasciae latae shows significant increases in the first half of the swing phase (60–80% of the gait cycle), and the tibialis anterior shows this in the second half of the swing phase (80–100% of the gait cycle). This behaviour can be observed in all test conditions (see also [1,2]).

For the flexor muscles (Rectus femoris, biceps femoris, and gastrocnemius lateralis—see Figure 2), a significant increase in muscular activity can be found in all test conditions for around 80–90.5% of the gait cycle, i.e., just before touchdown. These test conditions include all three patterns—the striped, Lisbon, and the random dots pattern—in the greyscale and coloured versions (see also [1,2]). An overview of all muscular data is provided in Figure 5.

For the extensor muscles (gluteus maximus, vastus medialis, and soleus), no such clear response emerges. Of the extensors, the soleus shows the largest deviation from the control condition, which manifests in a statistically significant increase in activity from 15–50% of the gait cycle, which is still in the stance phase. Again, in all conditions, the vastus medialis also shows a significant increase of around 40% of the gait cycle, while the activity of the gluteus maximus is slightly reduced during this time frame (see also [1,2]). The activity of the tensor fasciae latae significantly increased during the first half of the swing phase (60–80%). In contrast, the tibialis anterior is more active during the second half of the swing phase (85–100%).

These changes, which are present for all test conditions, point towards a control paradigm that is characterised by a shift towards the distal muscles in the extensors just before takeoff and an increase in the flexor activities, especially in the proximal muscles. The distal limb elements are known to be the first to react to disturbances [42], although the perturbations applied in most tests so far are mechanical. Lateral stabilisation during the swing phase is also affected, which manifests as an increase in activation of the tensor fasciae latae in the first half and the tibialis anterior in the second phase. This may contribute to lateral stabilisation [43], starting in the proximal limb and propagating towards the distal limb.

### 3.2. Lateral and Vertical CoM Deviation

**Figure 6:**
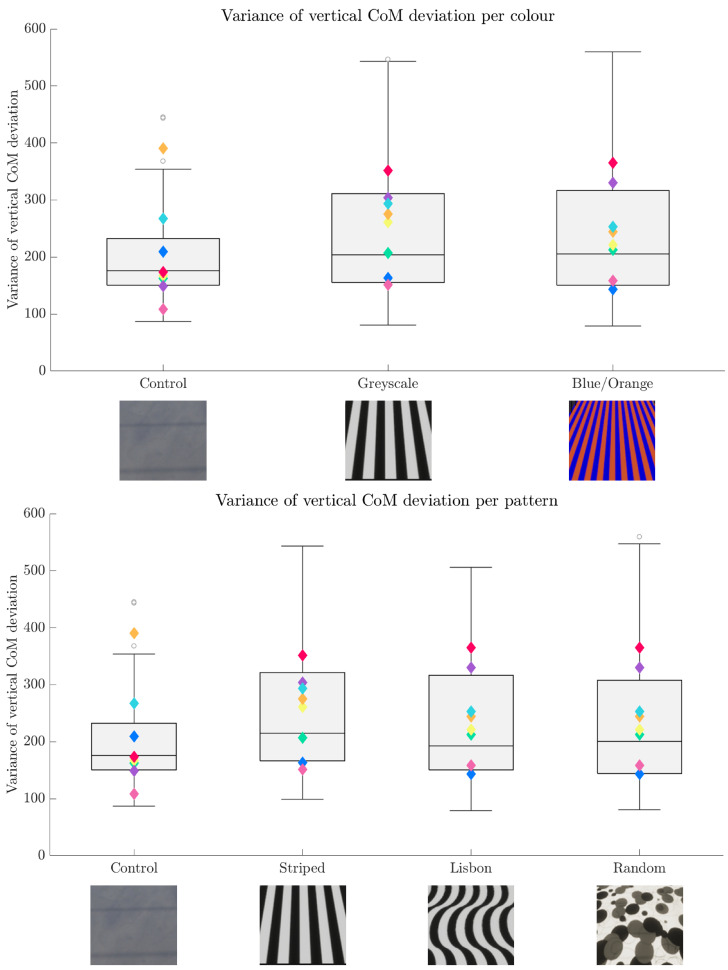
Boxplots of the variance of the vertical CoM deviation per colour condition (**upper**) and pattern (**lower**). The colourful diamonds represent the mean of each of the eight participants per condition. Especially the participants “red” and “purple” show a noticeably higher mean variance during the test conditions. The least variance is present in the control condition, while all the test conditions show higher variances.

**Figure 7:**
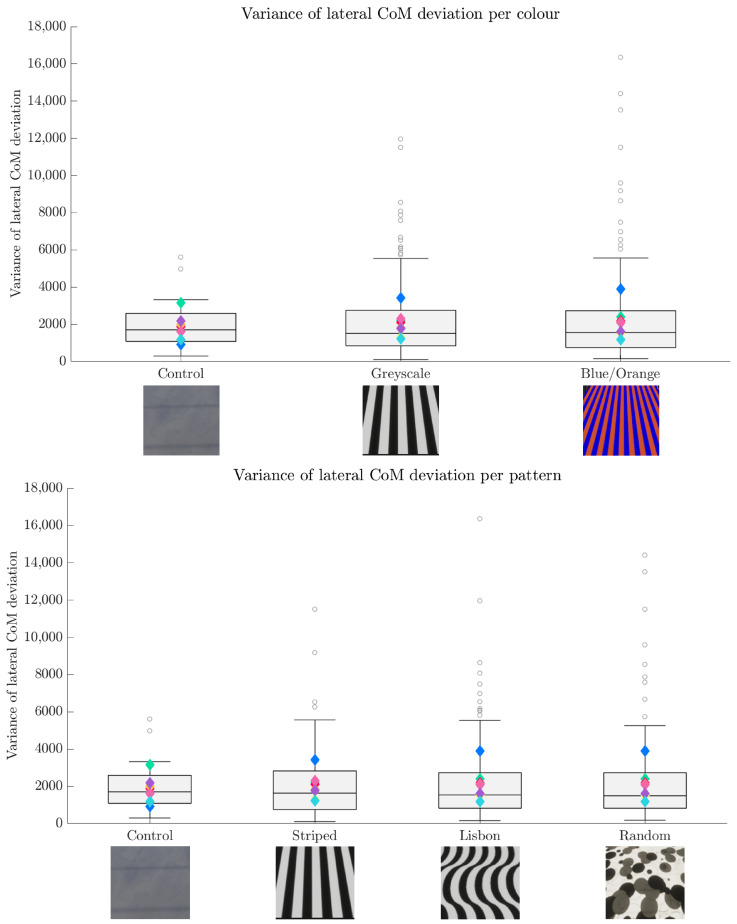
Boxplots of the variance of the lateral CoM deviation per colour condition (**upper**) and per pattern (**lower**). The colourful diamonds represent the mean of each of the eight participants per condition. In both variations, the variance does not change significantly between the conditions. However, the number of outliers is higher in all the test conditions. Additionally, notice that for the participant represented by blue, the mean variance increases notably in all test conditions.

The centre of mass (CoM) was approximated using the average of the motion capture data from the three pelvis markers. These CoM estimates were then clustered by condition and analysed using a linear mixed model (LMM) by using the pattern and the colour as fixed effects and the participant as random effect. The results are depicted in Figure 6 and Figure 7.

The statistical tests yielded no significant results for colour (β = 76.39; *SE* = 228.08; *p* = 0.73) or pattern (β = 104.57; *SE* = 183.30; *p* = 0.57) with respect to the lateral deviation of the CoM estimate. Moreover, no interaction between pattern and colour is present in the lateral direction (β = −35.38; *SE* = 128.89; *p* = 0.78).

In the vertical direction, a significant effect from the colour (β = 25.32, *SE* = 11.64, *p* = 0.03) has been identified. There was no statistically significant effect for the pattern (β = 14.54; *SE* = 9.19; *p* = 0.11) or the interaction between the colour and pattern (β = −11.12; *SE* = 6.55; *p* = 0.09). Thus, the statistical results yield a significant increase in vertical CoM deviation, which is attributed to the colour.

### 3.3. Uncomfortableness Metric

As expected, the Fourier-based metrics [40] yield the lowest (and, therefore, the best) scores for the control condition (see Figure 3). All versions of the metric punish a high-contrast environment. These results of the algorithm indicate that the striped pattern in greyscale is the worst pattern-colour combination in this experiment.

Since the colour metric from [41] sums the local colour contrast in the image, the coloured versions of the patterns always score highest (see Figure 4). Here, the coloured Lisbon pattern scores the lowest, while the greyscale striped pattern has the best score and is even better than the control condition. This is because the control condition was not plain white but light grey.

### 3.4. Comparison of the Metric to Human Data

The participants all reported that they disliked either the coloured or the greyscaled version of the Lisbon pattern the most. Although the self-reports agree with the colour metric’s estimate that the coloured versions are more uncomfortable (see Figure 4), the metric also judges the pattern of linear stripes to be less disturbing than random round shapes, but the participants favour the opposite. Since the metric is indifferent towards the shape of the pattern, it cannot offer an explanation of the participants’ preference for the random pattern.

## 4. Discussion

In order to understand how repetitive visual patterns influence gait, as reflected in biomechanical gait parameters, an experiment using three different patterns was conducted. Following [1,2,29], we expected an increase in the lateral and vertical deviation of the CoM when walking on these patterns. Furthermore, we expected changes in muscular activity that were consistent with a more careful gait. The data from eight participants showed significant increases in normalised muscular activity in the flexor muscles just before touchdown and in the soleus muscle during stance. Moreover, the vertical deviation of the CoM increased significantly. This indicates more careful movement and potentially adapted motor control strategy. Another supporting hint can be found in the increase in outliers of the lateral and the increase in higher variance scores of the vertical CoM deviations.

This adaptation might be regarded as a transition from stable to unstable movement. Stable movements can be modelled by neuromechanical and biomechanical models. One of these approaches uses a blending of reflex pathways, which is pro-prioceptive length (LFB), velocity (VFB), and force feedback (FFB) [44]. With the corresponding reflex gains, the so-called sensori-motor maps (SMM) can be calculated to understand the individual contribution of the sensory pathways during multisensory integration [44]. This model results in a certain area in which stable movements can be realised. Typically, such an area is mathematically compact. However, it is possible that the stable areas in these maps dissolve if certain conditions are met [45,46], which results in unstable walking. Classic SMMs focus on pro-prioceptive feedback pathways in a muscle [44]. Our results suggest that not only the pro-prioceptive feedback pathways considered so far but also the visual pathways have an influence on stable walking. The evaluation of an extended SMM model, including these visual feedback pathways, is left for future work.

The transition between a stable and unstable gait can be described not only by biomechanical gait parameters but can also be estimated by using the discomfort metric [40,41] used in this study. The results of the metric did align with an increase in vertical CoM deviation, which serves as an additional indication that repetitive patterns can influence human gait. However, the results of the algorithmic estimate only partly align with the self-reports of the participants. Although our participants indicated that they felt the Lisbon pattern was the most uncomfortable, it scored better in the Fourier-based metrics compared to the striped pattern (see Figure 3). One explanation could be that this metric does not consider the shape of the lines. The colour metric’s prediction, however, is in line with the human self-reports, with the Lisbon pattern scoring worst within the coloured patterns (see Figure 4). This could further be clarified if the participant’s self-reports were obtained on a rating scale. With this, both the algorithmic estimate and the self-reports could be compared quantitatively.

A possible next step would be to pinpoint the threshold where this dissolving into a noncompact solution occurs. This could be carried out by designing multiple versions of the same pattern (e.g., striped) while varying one singular parameter (e.g., the spatial frequency). When combined with the Fourier-based metric [40], this could be sufficient to find the amount of “repetitiveness” that is needed to dissolve the compact solution in the SMM.

This calls for architects and structural designers to consider visual clarity in structures. Since visual perturbations can lead to an inhibited gait, even in healthy young people, we would expect further inhibitions in the gait of humans with certain preconditions. This expectation is in line with previous findings [28]. This visual clarity is also encouraged by standards such as [47], which enforces the visual prominence of key features, e.g., handrails, to aid people with visual impairments while also fulfilling the needs of people without impairments.

### Limitations

While the biomechanical measurements were reliable in our study, there were issues with the VR system, which ultimately led to the exclusion of four participants’ datasets. These mainly manifested in grey screens during the trials, which most likely occurred due to computational limitations and the improper alignment of the WiFi link box of the wireless Vive system. After fixing these issues, the experiments continued as intended. It has to be noted that one of the excluded participants assumed that the grey screens were part of the experiment and, therefore, did not report it to the experimenter; the issue was discovered only after this participant had finished the runs. In general, the sample size was limited, mainly due to the high effort required to perform the experiments. Further, the sample is quite uniform with regard to age and general demographic backgrounds. Further study is needed to confirm whether the findings also translate to other groups of people, especially the elderly, patients with balance-related disorders, or people with different cultural backgrounds. For example, one could expect a more natural walking style from participants from Portugal or Brazil, where such patterns, called *calçada portuguesa* (e.g., [37]), are commonly used in pedestrian walkways. With that in mind, it has to be noted that the patterns used in this study were not chosen based on the literature but rather on observations within the real world. While possibly compromising the theoretical implications, this emphasises the importance of the practical use case.

Further limitations arise from the possible effects of adaptation. Since every condition was repeated five times, the later trials could differ from the first occurrences of each condition. However, this was countered by the randomised order of the conditions. This was not addressed in this analysis but could be of theoretical interest because there could be differences between pedestrians who are often moving in specific structured environments or only passing it occasionally.

Since it is not practical to collect ground reaction force (GRF) data using standard force plates, it is currently not possible to analyse the dynamics of the human body during VR walking. However, expanding the setup to be able to include GRF data is planned in the future using insoles containing force sensors.

## 5. Conclusions

Our results point toward a multitude of changes in biomechanical parameters caused by repetitive patterns, especially in the later stages of the gait cycle. Repetitive patterns can, indeed, lead to an adapted gait style, which is manifested in an increase in vertical CoM excursion and changes in muscular activation.

## Data Availability

The data are available upon request due to restrictions (ethical reasons).
